# Neuroprotection: The Emerging Concept of Restorative Neural Stem Cell Biology for the Treatment of Neurodegenerative Diseases

**DOI:** 10.2174/157015911795596603

**Published:** 2011-06

**Authors:** Barbara Carletti, Fiorella Piemonte, Ferdinando Rossi

**Affiliations:** 1 Unit of Neuromuscular and Neurodegenerative diseases, Children’s Hospital and Research Institute “Bambino Gesù”, Rome, Italy; 2 Scientific Institute of the Fondazione Cavalieri Ottolenghi; 3 Neuroscience Institute of Turin (NIT), University of Turin

**Keywords:** Neural stem cells, neurodegenerative disorders, neuroprotection, neurotrophic factors, oxidative stress.

## Abstract

During the past decades Neural Stem Cells have been considered as an alternative source of cells to replace lost neurons and NSC transplantation has been indicated as a promising treatment for neurodegenerative disorders. Nevertheless, the current understanding of NSC biology suggests that, far from being mere spare parts for cell replacement therapies, NSCs could play a key role in the pharmacology of neuroprotection and become protagonists of innovative treatments for neurodegenerative diseases. Here, we review this new emerging concept of NSC biology.

## INTRODUCTION

Although very different from one another, neurodegenerative diseases share common mechanisms, which lead to the dysfunction and loss of specific subsets of neurons. The general idea is that regardless of the pathogenic mechanism, neuronal death is induced by molecular cascades that may be common to multiple disorders. For instance, a characteristic feature recurring among many neurodegenerative diseases is the aggregation and misfolding of particular proteins. The affected proteins may differ in different diseases, but the way the neurons respond to overwhelming aggregates may converge towards a limited range of cell death pathways.

Oxidative stress, inflammation, mitochondrial dysfunction and defects of axonal transport are among the mechanisms that determine cell degeneration in most neurodegenerative disorders. Such commonalities among neurodegenerative diseases are particularly appealing, not only because they may unravel some general principles governing the biology of neuronal death, but also because they may allow the development of efficient therapeutic approaches for different pathological conditions.

At present, the available treatments for neurodegenerative diseases are inadequate. They are generally designed to counteract symptoms, but they are ineffective in reversing the progression of the disease. In this scenario, the possibility to increase neuronal survival by interfering with cell death processes emerges as a promising strategy to treat different neurodegenerative disorders. Once identified and tested for their efficacy, neuroprotection procedures might be applied either in patients with incipient clinical signs or, as a preventive approach, in individuals that have high risk of developing the disease in the future.

From a biological standpoint a neurocytoprotective effect could be achieved through several different ways, such as antagonizing the cytotoxic process triggered by the pathogenic event (e.g. oppose oxidative stress or inflammation-related processes), or reinforcing endogenous protective systems (e.g. production of neurotrophic factors, antioxidant substances, etc.).

To date, the strategies applied to discover effective neuroprotective agents have been focused on single pathways and only achieved partial results, thus suggesting that the concept of neuroprotective treatment should be revised. Indeed, multiple factors contribute to degenerative processes and, therefore, it would be better to consider either multiple treatments targeting different cell death cascades, or single agents that moderate multiple cell death pathways. In the last few years a novel therapeutic opportunity emerged from stem cell biology. A series of neural transplantation studies highlighted unexpected neuroprotective actions exerted by transplanted neural stem cells on dysfunctional host neurons. These neuroprotective properties of NSCs appear as the expression of an interesting developmental program. The study of the mechanisms involved in this program and the potential use of exogenus NSCs as therapeutic “tools” are indubitably very promising and open a new way for future neuroprotective therapy. Here, we will describe this novel application of NSCs for the treatment of neurodegenerative diseases and discuss some of the related mechanisms.

## STEM CELLS: EVIDENCE FOR NEUROPROTECTION

Several studies have shown that undifferentiated NSCs might achieve functional effects in animal models of neurological disorders following transplantation [1], but a most interesting aspect of these reports is that functional improvement results from induced self-repair and neuroprotective effects rather than cell replacement.

The notion that NSCs are able to rescue dysfunctional neurons was first proposed in 2002 in two studies, dealing with Parkinson’s disease [2] and spinal cord injury [3]. Ourednik and colleagues [2] showed that transplantation of NSCs is able to preserve the function of degenerating DA neurons following systemic injection of MPTP in the mouse. After drug intoxication, the animals underwent unilateral implantation of NSCs, which rescued the expression of tyrosine hydroxylase and dopamine transporter in the ispilateral host DA neurons. In the work by Teng and colleagues [3], a similar neuroprotective effect was observed by implanting a polymer scaffold seeded with NSCs in a model of traumatic spinal cord injury. Animals implanted with scaffold containing NSCs in the emisectioned spinal cord showed long-term improvement of motor function that could be related to reduction of secondary degeneration. The functional recovery was exerted by the scaffold itself that hampered the formation of glial scar, but also by the donor NSCs that antagonized excitotoxic mechanisms. This effect preserved neurons and oligodendrocytes from secondary degeneration and enhanced regenerative processes. 

Following these pioneer works, several studies reported neuronal protection as the therapeutic target of NSCs transplantation, and suggested that this property of NSCs could be applied to a wide range of neurodegenerative disorders. Hence, a neuroprotective action mediated by NSCs has been reported in models of multiple sclerosis [4], amyotrophic lateral sclerosis [5], stroke [6], Purkinje cell death [7] and retinal degeneration [8]. In a mouse form of chronic inflammation, the R-EAE, which mimics aspects of human multiple sclerosis, it was shown that grafted NSCs promote an immune-like response that result in a long-lasting neuroprotection [4]. NSCs derived from the adult telencephalic subventricular zone, and intravenously injected in treated mice, were recruited around inflamed blood vessel of the deep CNS parenchyma where they induced the programmed death of infiltrating T cells. This mechanism reduced the extent of the neurodegeneration caused by the repeated inflammatory episodes of the R-EAE. A similar result was obtained with human embryoid body derived cells in a virus induced motor neuron disease [9]. EBD cells transplanted into the lumbar cerebrospinal fluid migrated into the damaged tissue, probably responding to environmental attractive cues, and reduced motor neurons death, thus promoting functional recovery. The general emerging principle is that grafted NSCs act on the microenvironment around dying neurons and create conditions that favour their survival. In an elegant study, Clement and collegues [5] confirmed this idea of neuroprotection creating a chimeric model of ALS. They generated mice that have both cells with a superoxide dismutase-1 mutation and wild type cells and demonstrated that within the spinal cord motor neurons carrying the mutation were protected by the surrounding wild type cells, presumably astrocyte progenitors.

NSCs-mediated host cell rescue and functional recovery was also shown in nervous (nr) mutant mice [10], in which a subset of Purkinje cells degenerate at the end of development. NSC transplantation into the cerebellum of newborn mutant mice prevented the loss of host PCs [7]. Similarly, subretinal injection of NSCs in a rat model of retinitis pigmentosa and secondary photoreceptor degeneration preserved retinal activity and function [8].

The molecular mechanisms responsible for the NSC neuroprotective action are not fully understood, but likely reside in the NSC ability to produce a wide repertoire of neuroprotective substances and trophic factors. NSCs constitutively produce and secrete neurotrophic factors both *in vitro* and *in vivo* and maintain this property once grafted into the diseased CNS [11]. Thus, the NSC therapeutic effect showed in a significant number of different cases could be attributable to the release of specific soluble factors. Grafts of human HB1.F3 NSCs (a fetal-derived stem cell line) attenuate parkinsonian motor symptoms in 6-hydroxydopamine-lesioned rats, and this effect is mainly attributable to the secretion of human recombinant SCF [12]. Similarly, EBD cells protect motor neurons from degeneration in rats paralyzed after infection with neuroadapted Sindbis virus, likely through the production of diffusible growth factors TGF-α and BDNF [9]. Finally, transplanted NSCs (mouse clone C17.2) support the growth of motor and sensory axons in the injured spinal cord by releasing BDNF, NGF and GDNF [11]. The ability to release factors that promote a significant recovery of function in damaged neural tissue seems also common to some other stem cell types, such as for example mesenchymal stem cells [for reviews see 13 and 14].

Studies on ischemic stroke revealed that transplanted neural progenitor cells exert a therapeutic neuroprotection by stimulating anti-inflammatory, glial scar inhibitory and anti-apoptotic mechanisms. A significant reduction of mRNA levels of pro-inflammatory and apoptotic mediators was registered in the lesioned area after colonization by NPCs and, in particular, a down-regulation in the expression of the cytokines TNF-α and IFN-γ has been suggested to account for the increased neuronal survival in the ischemia affected regions of transplanted mice [15]. Furthermore, a direct protection from apoptotic cell death *via* caspase activation has been for example demonstrated for NGF and BDNF providing evidence for a survival promoting mechanism exerted by NSC growth factors secretion [16]. Nevertheless, the production of diffusible factors might be not the only mechanism involved in NSC rescue of imperilled endogenous neurons. Recent studies showed that a pivotal role in the dynamic of neuroprotection is played by the direct contact between NSCs and host neurons *via* the formation of gap junctions; this NSC coupling could not only deliver protective factors, but might be also implied to remove toxic molecules, for example interacting with reactive glial cells [17]. 

## NEUROPROTECTIVE PHENOTYPE OF THE “STEM CELL” STATE

The studies mentioned above have shed a new light on the ways stem cells promote neuronal recovery by protecting damaged neurons from degenerative mechanisms. Such neuroprotective capabilities of NSCs intervene at multiple levels, so that their beneficial effects can be obtained in different pathologic conditions. Furthermore, these properties appear to be active regardless of the NSC species, their source or the methods of expansion and propagation [18]. How do NSCs exert therapeutic effects in such different conditions? Which properties are responsible for these effects? The evidence provided by the available literature in the field clearly indicates that the rescue of damaged host neurons is due to donor NSCs that remain undifferentiated in a “stem cell” state. Following transplantation only a minority of the donor NSCs differentiate in mature neurons. Such a restricted fraction of differentiated elements, even if they succeed in integrating into the host circuitries, cannot account for the significant therapeutic effects that are frequently observed in these conditions. Indeed, the grafts usually contain a pool of undifferentiated nestin-positive cells that might be the actual effectors of long-lasting beneficial effects on the pathological processes [3, 2, 11].

Evan Snyder and colleagues proposed that the neuroprotective properties of NSCs could be an aspect of their fundamental biology [19]. The “stem cell” state could constitutively express an intrinsic developmental program, which may involve an enhanced resistance to environmental stress. Indeed, these studies highlight the improved “vigilance” of NSCs with respect of endogenous neurons [20]. Hence, to realize their therapeutic effect NSCs have to be strongly resistant to the hostile environment of the diseased CNS. This is particularly evident when NSCs are transplanted in a toxic milieu, such as that of CNS tissue affected by a metabolic disorder. NSCs grafted in a mouse model of Krabbe leukodistrophy, characterized by accumulation of the toxic glycolipid psichosine, were able to resist to the toxic microenvironment and made cell replacement possible in a milieu that would be otherwise prohibitive for donor cells [21]. However, the most important outcome of this protective action of NSCs is that they are able to provide the neighbouring mature neurons with the ability to cope with the damage-perturbation caused by the pathology. For instance, the work of Lu and collegues [11] reveals an internal cross-talk among the NSC-derived cell population, in which immature cells provide trophic support to their daughter cells committed to become mature neurons. In this scenario, the adjacent host neurons would draw indirect benefit from such NSC-derived trophic support. It has been reported that within a NSC clone non-neuronal cells produce growth factors, such as GDNF, whereas only their neuronal progeny expresses the cognate receptor. This phenomenon indicates that stem cells are endowed with particular properties aimed at providing support to newborn neurons. This system appears as a sort of mother’s care-taking mechanism, in which undifferentiated stem cells protect their descendants that have engaged in phenotype acquisition, but still require external support to complete their maturation and meaningful integration in the neural tissue.

## THE OXIDATIVE STRESS EXAMPLE

In this last section of the review we will discuss the neuroprotective properties of NSCs considering a particular type of cell damage, involved in many neurodegenerative diseases, i.e. oxidative stress. Oxidative stress is caused by an imbalance between the production of reactive oxygen species and the cellular antioxidant defence, which is usually able to detoxify the reactive intermediates and repair the resulting damage. Evidence has been accumulating that oxidative stress is one of the most common molecular mechanisms responsible for the pathogenesis and progression of several neurodegenerative diseases. Mitochondrial dysfunction and oxidative damage to tissue have been described in Parkinson’s disease [22-25], Huntinghton’s disease [23, 24], Amyotrophic lateral sclerosis [23, 24, 26], Friedreich’s ataxia [27-31], Hereditary spastic paraplegia [23, 24, 26] and Alzheimer's disease [23, 24, 33]. Therefore, we will first ask whether the above described phenomena of stem cell-mediated neuroprotection can also apply to the case of oxidative stress and, consequently, whether stem cells can be exploited to counteract this type of neuronal damage.

It has been demonstrated that NSCs and their postmitotic progeny differ in their redox state [34]. The “stem” cell phenotype, because of its self-renewal capacity, is associated with a more reduced intracellular environment compared to postmitotic neurons. This phenomenon results from a precise mechanism, in which the redox state of a stem/progenitor cell emerges as a biochemical regulator between self-renewal and differentiation. Recently, Madhavan and colleagues [20] postulated that the cellular redox condition of NSCs and their differentiated progeny also change the ability to defend themselves against oxidative stress. These authors demonstrated that NSCs have lower content of intracellular ROS and higher basal levels of several antioxidant molecules, so that they are more resistant to oxidative insult than postmitotic neurons. NSCs are not only better equipped against oxidative stress, but are also able to react more efficiently against this dangerous insult by increasing the synthesis of antioxidant proteins. Together, these findings indicate that stem cell “vigilance” is active and potentially beneficial in the occurrence of oxidative stress. But, are NSCs able to transmit this increased “vigilance” towards oxidative stress also to neighbouring mature neurons? Could be this specific antioxidant competence used for a therapeutic purpose in neurodegenerative diseases? Indeed, Madhavan and colleagues [35] showed that NSCs exert an antioxidant neuroprotective mechanisms towards the surrounding neurons. These authors applied the oxidant drug 3-NP to the neuronal microenvironment and demonstrated the protective effect exerted by NSCs either co-cultured with primary neurons *in vitro* or grafted to the striatum *in vivo*. These experiments showed that NSCs promote an effective antioxidant response in the surrounding neuronal cells that usually would not resist to the oxidative insult. The effect involves the release of growth factors that directly modulate the activity of antioxidant defence mechanisms. Specifically, secretion of CNTF and VEGF correlates with the upregulation of SOD2 both in NSCs themselves and in the surrounding cells. A similar antioxidant modulator activity has been demonstrated also for MSCs [36]. These results lead many authors to envision a NSC process in which the secretion of growth factors or neuroprotective molecules is adapted to the pathologic environment to promote survival of affected neurons. In fact, the behaviour of NSCs grafted into a diseased nervous system seems regulated by a series of graft-host interactions, which lead NSCs not only to adjust their migration and distribution, but also to regulate the secretion of trophic factors to the requirement of the recipient tissue.

The cross-talk between NSCs and dysfunctional neurons may offer the opportunity to intervene with new treatments that favour this interaction. For example, it has been demonstrated that the increased expression of the adhesion molecule L1 in MPTP lesioned mice enhance the benefits derived from NSCs grafts. L1 act in the nervous system during developmentt and its *de novo* expression in an adult dysfunctional nervous system may promote those cross-talk mechanisms normally present during development between NSCs and newborn neurons [37].

## CONCLUDING REMARKS

The articles reviewed here highlight a new concept of stem cell “restorative biology”. Part of the intrinsic developmental program of stem cells seems to constitutively activate protective and reparative processes in response to specific environmental demands. This neuroprotective stem cell property is mostly mediated through the release of specific trophic factors that modulate the survival capabilities of surrounding neurons. Such a notion may well open new perspectives for the treatment of numerous degenerative conditions.

## ABBREVIATIONS

NSCs: = Neural Stem Cells
(MPTP: = 1-methyl-4-phenyl-1,2,3,6 tetrahydropyridine
ALS: = Amyotrophic lateral sclerosis
CNS: = Central nervous system
R-EAE: = Relapsing remitting experimental autoimmune encephalomyelitis
EBD: = Embryoid body derived
PCs: = Purkinje cells
SCF: = Stem cell factor
BDNF: = Brain derived neurotrophic factor
NGF: = Nerve growth factor
GDNF: = Glial derived neurotrophic factor
MSCs: = Mesenchymal stem cells
NPCs: = Neural progenitor cells
TNF-α: = Tumor necrosis factor α
IFN-γ: = Interferon-gamma
ROS: = Reactive oxygen species
3NP: = 3-nitropropionic acid
CNTF: = Ciliary neurotrophic factor
VEGF: = Vascular endothelial growth factor
SOD2: = Superoxide dismutase-2
